# Assessment of Changes in Lipids Metabolism in Patients with Degenerative Joints and Discs Diseases Subjected to Spa Therapy

**DOI:** 10.1155/2019/4732654

**Published:** 2019-07-24

**Authors:** Michał Kasperczak, Jadwiga Kuciel-Lewandowska, Jan Gnus, Małgorzata Paprocka-Borowicz

**Affiliations:** Department of Physiotherapy, Medical University of Wroclaw, Poland

## Abstract

**Introduction:**

High levels of total cholesterol, triglycerides, and, connected with them, lipoprotein fractions may result in atherosclerosis. There are various forms of therapy used to prevent cardiovascular diseases, such as balneophysiotherapy, the effectiveness of which is confirmed by numerous scientific publications.

**Objective:**

The objective of this study was to assess the impact of balneophysiotherapeutic procedures on the systemic metabolism of lipids in patients suffering from osteoarthritis of the motor organ.

**Material and Methods:**

The study was conducted in the Health Resort Świeradów-Zdrój. Observation included patients undergoing radon water therapy. Before therapy and after 21 days of treatment, lipid profile was assessed with the use of standard colorimetric assay. Study group consisted of n=34 patients with degenerative joints and disc disease. The mean age of patients was 56.5l. The control group consisted of 17 people selected among the employees of the spa also suffering from osteoarthritis. The mean age was 54.2 years.

**Results:**

The results of the study are based on a single, 21-day health resort stay period in April/May. A statistically significant increase in HDL cholesterol levels was observed in female patients having undergone health resort treatment (*P*<0.01). Statistically significant drops in LDL cholesterol and TG levels were observed in the control group (*P*<0.01). An increase in HDL levels was observed in the male and female control subjects, with* P*<0.05.

**Conclusions:**

(1) After the end of therapy, there were no changes in lipid metabolism in men, while in the group of women an increase in HDL level was observed. (2) In the control group, statistically significant changes in the field of lipid metabolism may be related to lifestyle changes as a result of educational activities conducted prior to the research. (3) Due to the divergent results, it is advisable to conduct randomized studies in a larger population. This trial is registered with NCT03274128.

## 1. Introduction

Balneotherapy in Europe, being a heritage of Roman and Ottoman empires, is a recognized medicinal technique in numerous European countries such as Germany, Italy, Hungary, or France. It is also an established therapeutic modality in certain Asian countries (Japan, Korea, and Taiwan) featuring geological formations with postvolcanic activity [1]. Besides being based on the physical properties of water, balneotherapy involves mineral substances being absorbed through the skin, mucosal membranes, and airways [2]. In recent decades, an increasing number of studies were published to confirm the efficacy of health resort treatments [3].

High levels of total cholesterol, triglycerides, and related lipoprotein fractions play a decisive role in the development of atherosclerosis. However, atherosclerosis is a multifactorial disorder which may only partially (i.e., in about 50%) be attributed to risk factors including smoking, hypertension, age, gender, hypercholesterolemia, and hypertriglyceridemia.

## 2. Objective

The objective of this study was to assess the impact of balneophysiotherapeutic procedures on the systemic metabolism of lipids in patients suffering from osteoarthritis of the motor organ.

## 3. Material and Methods

Observation was carried out in patients undergoing health resort therapy as a part of a 21-day stay period during the spring season. Venous blood was collected from patients before the treatment as well as 18 days into the treatment. Heparinized plasma was used for lipid profile assessments. Biological material was collected using a disposable, sterile, closed-system instrument. After collection, samples at the temperature of 6°C were transported into the lab for analysis. Standard, commercially available assays were used.

The study was carried out according to a nonrandomized design. The study group consisted of n=35 patients with joint or spinal pain due to osteoarthritis or discopathy. The age range was 47-63 years, with the mean age of 56.5 years. The group consisted of 22 female and 13 male patients. The selection criteria included the established diagnosis of osteo- and/or spondyloarthritis, age in the range of 45-65 years, consent to participate in the study, and lack of contraindications for comprehensive health resort treatment. Exclusion criteria included the lack of consent for participation in the study, age below 45 or above 65 years, and presence of disorders constituting contraindications for the treatment (according to the standard list of indications and contraindications for health resort treatment) as well as the presence of metabolic disorders. Therapeutic radon-active water was used in the treatment. Treatment measures included whole body immersion radon baths, with temperature of 37°C and duration of 15 min., administered every second day and oral inhalations of radon, with temperature of 37°C and duration of 15 min., administered every second day. Baths and inhalations were delivered on alternate days. A total of 15 radon procedures were delivered during the resort stay period. In addition, the treatment consisted of peat compresses, kinesiotherapy, and physical therapy. Patients were prescribed with a series of 10 procedures of particular types depending on their physical status and complaints. An example set of treatment procedures included radon water baths, radon inhalations, peat compresses, group and individual gymnastics, laser biostimulation, and interference currents.

Presented below are the dosage regimens for individual treatments:

- radon-containing water baths with whole body or partial upper and/or lower limb immersion at the temperature of 37-38°C for the duration of 20 minutes;
- oral inhalations of radon for the duration of 15 minutes, every second day;
- peat treatments (peloid therapy): partial peat compresses at the temperature of 40-42°C for the duration of 20 minutes;
- therapeutic gymnastics in a therapeutically neutral water pool;
- individual apparatus-based and group gymnastics; exercises were customized to each patient considering their individual fitness; the mean duration of kinesiotherapy was 30-45 minutes;
- outdoor treatment: walks, outdoor motion classes;
- dry massage: depending on the needs, massage was delivered to the cervical (CC), thoracic (TH), or lumbar spine (LS);
- laser therapy: parameters: sweeping technique, continuous operation, wavelength 808 nm, 12.0 J power, 400 mV, duration 30 s;
- low-frequency magnetic field: duration 20 min., square pulse, 5 mT induction, frequency 20-50 Hz;
- ultrasound therapy: parameters: 800 kHz/6 cm^2^ probe, pulse wave consisting of ultrasound pulses of 2 ms at 9 ms intervals, dose range 0.5-0.6 W/cm2, duration 6 minutes;
- cryotherapy: ventilation, duration 2-3 minutes, temperature from −80°C to −110°C;
- electrotherapy: Bernard's biodynamic currents parameters: DF1 CP4 LP4; Nemec's interferential currents (frequency range 0-100 Hz); transcutaneous electrical nerve stimulation (TENS); square-wave pulse current, pulse width 0.2 ms, frequency 40 Hz, intensity regulated within the 0-100 mA range;
- light therapy: Sollux blue filter lamp, irradiation distance 30-40 cm, duration 15 min; Bioptron lamp, irradiation distance 10 cm, duration 5-10 min.

The study treatment took advantage of radon-active waters of the Świeradów-Zdrój Health Resort which have been used for therapeutic purposes for more than one hundred years. The waters are characterized by low mineral content, and their main therapeutic factor consists in their radon activity of 303.1-441.5 Bq/L. Alpha radiation measured within the treatment facilities (inhalatorium, bath cabins, and pool) was in the range of 295,44-720,98 × 10^−13^ J. The measurement permits evaluation of patients' exposure. The dose of absorbed radiation was not determined since the radioactivity was a variable parameter. It depended on body composition, particularly on the content of adipose tissue and absorption area, concomitant diseases, as well as operational radiation losses. Measurements within the treatment facilities were taken daily using certified detectors. Every 3 months, the measurement results are analyzed in the Department of Radiological Protection of the Nofer Institute of Occupational Medicine in Łódź.

The anti-inflammatory, desensitizing, and analgesic effects of radon are attributed to stimulation of the adrenal cortex and increased production of steroid hormones. Studies revealed increased serum levels of luteinizing hormone, growth hormone, cortisol, testosterone, estradiol, and estriol. Radioactive treatment improves peripheral circulation, reduces swelling as well as joint and musculotendinous pain, and improves motor output. Other findings include reduced arterial blood pressure, cholesterol and triglyceride levels, reduced erythrocyte sedimentation rates, increased hemoglobin levels and erythrocyte counts, and increased levels of ionized calcium, parathyroid hormone, calcitonin, as well as increased rate of elimination of harmful metabolites [4–10].

A control group was also provided for the study design. It consisted of 17 individuals selected from among the resort personnel and included 11 female and 6 male subjects aged 50 to 62 years, with the mean age of 54.2 years. Subjects enrolled in the control group were also burdened by osteoarthritis of the motor organs while not taking advantage of the treatment facilities of the resort (i.e., not exposed to radon). The main selection criteria included the established diagnosis of osteo- and/or spondyloarthritis, age in the range of 45-65 years, consent to participate in the study, and lack of contraindications for treatment. The inclusion and exclusion criteria in the control group were the same as in the study group.

Due to the fact that the concentration ranges differed for individual lipid profile assessments between both genders, the results obtained in both groups were divided into same-gender subgroups.

Statistical analyses were carried out using the Statistica 13 software package (StatSoft, Inc., USA). Arithmetic means, medians, standard deviations, and variability ranges (extreme values) were calculated for measurable variables. All quantitative variables were tested for the type of distribution using the Shapiro-Wilk test. Results obtained in the study and the control group were compared using the Mann-Whitney's U-test. In-group comparisons of results obtained in measurements I and II were carried out by means of unifactorial analysis of variance (ANOVA). The significance level for all comparisons was established at *α*=0.05; the P values were rounded to 2 decimal places.

## 4. Results

Systemic lipid profile reflects the systemic metabolism of lipids including the course of the metabolism of lipoproteins and cholesterol. The reference ranges of individual profile components are as follows:

1. total cholesterol: below 190 mg/dL (5.0 mmol/L);
2. HDL cholesterol: above 40 mg/dL (1.0 mmol/L) in males, above 46 mg/dL (1.2 mmol/L) in females;
3. LDL cholesterol: below 111 mg/dL (3.0 mmol/L);
4. triglycerides (TG): below 150 mg/dL (1.7 mmol/L).

Changes in lipid metabolism were observed in the study group as well as the control group. A statistically significant increase in HDL cholesterol levels was observed in female patients having undergone health resort treatment (*P*<0.01, Table 3). Statistically significant drops in LDL cholesterol and TG levels were observed in the control group (*P*<0.01) (Table 1). An increase in HDL levels was observed in the male and female control subjects, with* P*<0.05 (Tables 2 and 3).

## 5. Discussion

The results of the study are based on a single, 21-day health resort stay period in April/May. According to some authors, the time of the year may have a significant impact on the study outcomes. Statistically significant seasonal variability was observed for total cholesterol (*P*<0.02) and LDL cholesterol (*P*<0.01), with the highest levels being observed in winter and the lowest levels being observed in spring. No seasonal fluctuations were observed for HDL and TG levels. The largest drops in total cholesterol levels were observed in fall whereas the lowest drops were observed in spring [11].

In this study, a reduction in HDL cholesterol levels was observed in male patients in the study and the control group as well [12]. A statistically significant increase in HDL cholesterol levels was observed in female subjects. Oláh et al. also demonstrated increased HDL cholesterol levels in the study as well as in the control group. Their study, however, was conducted in a significantly smaller group with the majority of subjects being female and the study population not being subdivided according to the gender.

In this study, both male and female subjects were undergoing the same therapy and followed similar dietary regimens. We believe that the differences in the results may be due to the differences in the distribution of the adipose tissue in women and men [13].

A slight drop in LDL cholesterol levels was observed following the health resort treatment; however, the difference was not statistically significant. The result is confirmed by numerous publications based on studies conducted in subjects free of metabolic disorders. Oláh et al. were among the few groups of researchers who observed a statistically significant reduction in LDL levels after two weeks of balneotherapy. However, the drop in LDL levels returned to the nonsignificant range at treatment completion. [14].

Studies by Naumann et al. or Kamioka et al. revealed nonsignificant reduction in TC and TG levels [15, 16]. As shown by Li Xu et al., a significant drop in triglyceride levels (*P*<0.05) is observed in the study group while the cholesterol levels remain essentially unchanged [17]. Fioravanti et al. observed statistically significant reductions in TC and TG levels in diabetic patients with no statistical significance being observed for nondiabetic patients. Statistically significant reductions in LDL cholesterol levels were observed at treatment completion in both groups (*P*<0.05) [18].

Moser el al. observed a marked reduction in TC and HDL levels. A total of 61, mainly cardiovascular, patients were divided into 2 treatment groups. In addition to regular balneotherapeutic regimen, one group received crenotherapy (drinking therapy with chloride/sodium/iodine water) while the control group received isotonic sodium chloride solution. Low-fat diet was used throughout the treatment [19].

In their study of obese diabetic patients undergoing health resort treatment, Szafkowski et al. observed that the body weight loss achieved as a result of the treatment was accompanied by reduced levels of total cholesterol, LDL cholesterol, and triglycerides. A significant increase in HDL cholesterol levels was observed in patients with low baseline values [20]. In a study conducted at the underground “Wieliczka” Salt Mine Health Resort, nonsignificant reduction in TC and TG was observed in 80% and 66% of subjects, respectively. A statistically significant increase in HDL levels was observed in 73.3% of health resort patients [21]. A similar study was carried out by Goszcz et al. The study group consisted of patients with elevated blood cholesterol and triglycerides levels and Fontaine stage II and III extremity artery disease. Chloride/sodium, bromide, iodide, boron, and sulphide waters were used in the observation stage. One group of patients received water obtained directly from the spring while the other group received water that had been diluted by a factor of two. Sulphide water was administered 3 times a day before meals, with low-fat dietary regimen being followed. Statistically significant reductions in triglycerides and total cholesterol levels were observed in subjects receiving nondiluted water. No statistical significance was observed in the other group [22]. Health resort-based study by Włodarczyk revealed no changes in total cholesterol and triglyceride levels. Treatment included carbonic acid full-body immersion baths and whirlpool shank massage [23].

With no doubt, differences observed in the results are related to the types of treatment procedures and disorders being treated in patients. As reported in studies cited above, the lipid profiles tend to be more sensitive to balneotherapy in patients with atherosclerosis, type 2 diabetes mellitus, obesity, or lipid metabolism disorders.

Treatments involving the use of medicinal waters may lead to different metabolic outcomes due to the composition-related differences. Clinical improvement is usually observed while marked discrepancies may be observed in individual parameters. Therapeutic waters contain various elements and compounds involved in different metabolic pathways. For example, our own study conducted in the same group of patients revealed an increased total antioxidant status (TAS) in patients after treatment completion [24]. The increase in TAS value is indicative of enhanced production of antioxidants, including HDL cholesterol considered to be a member of this group [25, 26]. However, the small extent of the reduction in LDL cholesterol levels as well as of the increase in TC and TG levels despite the increasing TAS and HDL values remains unexplained.

Due to the observed discrepancies, the assessment of the impact of balneotherapy on the changes in lipid metabolism requires further studies being conducted in large groups of patients, preferably using isolated treatment modalities.

## 6. Conclusions

1. After the end of therapy, there were no changes in lipid metabolism in men, while in the group of women an increase in HDL level was observed.
2. In the control group, statistically significant changes in the field of lipid metabolism may be related to lifestyle changes as a result of educational activities conducted prior to the research.
3. Due to the divergent results, it is advisable to conduct randomized studies in a larger population.

## Figures and Tables

**Table 1:** Lipid profile changes after balneophysiotherapy (mean ± SD, mmol/L).

		TC	LDL	TG
Study group N=35	BEFORE	12.5 ± 1.4	7.5 ± 1.6	8 ± 4.2
	AFTER	12.7 ± 1.6	7.3 ± 1.6	8.3 ± 5.5

Control group N=17	BEFORE	13.8 ± 1.8	8.2 ± 1.9	7.6 ± 3.8
	AFTER	13.2 ± 1.6	7.9 ± 1.6 *∗∗*	6.8 ± 2.7 *∗∗*

*∗*P<0.05, *∗∗*P<0.01 compared to pretreatment values. TC: total cholesterol; LDL: low density lipoprotein; TG: triglycerides.

**Table 2:** HDL cholesterol level changes in male patients after balneophysiotherapy (mean ± SD, mmol/L).

		HDL
Study group N=13	BEFORE	3.3 ± 0.6
	AFTER	2.9 ± 0.4

Control group N=6	BEFORE	4.6 ± 0.9
	AFTER	3.5 ± 0.5*∗*

*∗*P<0.05, *∗∗*P<0.01 compared to pretreatment values. HDL: high density lipoprotein.

**Table 3:** HDL cholesterol level changes in female patients after balneophysiotherapy (mean ± SD, mmol/L).

		HDL
Study group N=22	BEFORE	3.6 ± 0.6
	AFTER	4.0 ± 0.8*∗∗*

Control group N=11	BEFORE	4.3 ± 0.7
	AFTER	4.1 ± 0.78*∗*

*∗*P<0.05, *∗∗*P<0.01 compared to pretreatment values. HDL: high density lipoprotein.

## Data Availability

The data used to support the findings of this study are available from the corresponding author upon request.
